# Rogue waves in the two dimensional nonlocal nonlinear Schrödinger equation and nonlocal Klein-Gordon equation

**DOI:** 10.1371/journal.pone.0192281

**Published:** 2018-02-12

**Authors:** Wei Liu, Jing Zhang, Xiliang Li

**Affiliations:** 1 College of Mathematic and Information Science, Shandong Technology and Business University, Yantai, 264005, P. R. China; 2 Chuo College of Technology, Tokyo, 114-8543, Japan; China University of Mining and Technology, CHINA

## Abstract

In this paper, we investigate two types of nonlocal soliton equations with the parity-time (*PT*) symmetry, namely, a two dimensional nonlocal nonlinear Schrödinger (NLS) equation and a coupled nonlocal Klein-Gordon equation. Solitons and periodic line waves as exact solutions of these two nonlocal equations are derived by employing the Hirota’s bilinear method. Like the nonlocal NLS equation, these solutions may have singularities. However, by suitable constraints of parameters, nonsingular breather solutions are generated. Besides, by taking a long wave limit of these obtained soliton solutions, rogue wave solutions and semi-rational solutions are derived. For the two dimensional NLS equation, rogue wave solutions are line rogue waves, which arise from a constant background with a line profile and then disappear into the same background. The semi-rational solutions shows intriguing dynamical behaviours: line rogue wave and line breather arise from a constant background together and then disappear into the constant background again uniformly. For the coupled nonlocal Klein-Gordon equation, rogue waves are localized in both space and time, semi-rational solutions are composed of rogue waves, breathers and periodic line waves. These solutions are demonstrated analytically to exist for special classes of nonlocal equations relevant to optical waveguides.

## Introduction

Since Bender and Boettcher [1] showed that in the spectrum of the Hamiltonian, large amounts of non-Herimitan Hamiltons with Parity-time-symmetry (*PT*-symmetry) possess real and positive spectrum, the *PT*-symmetry has been an interesting topic in quantum mechanics and has significant impact. In general, a non-Hermitian Hamiltonian *H* = ∂_*xx*_ + *V*(*x*) is called *PT*-symmetric if *V*(*x*) holds for *V*(*x*) = V*(−*x*). If set *V*(*x*, *t*) = *p*(*x*, *t*)p*(−*x*, *t*) in the Hamiltonian *H* above, then the Schrödinger equation *ip*_*t*_ = *Hp* is *PT*-symmetric. In recent years, many works on *PT*-symmetry have been presented [2–6]. *PT*-symmetry has been widely applied to many areas of physics, such as optics [4, 7, 8], such as Bose-Einstein condensates [9], such as quantum chromodynamics [10], and so on.

In [11], Ablowitz and Musslimani introduced the nonlocal nonlinear Schrödinger equation

$$\begin{array}{c} \begin{array}{cc} & iq_{t}\left(x,t\right)+q_{xx}\left(x,t\right)+2q\left(x,t\right)q^{*}\left(-x,t\right)q\left(x,t\right)=0 \end{array} \end{array}$$ (1)

and got its explicit solutions by inverse scattering. Quite a lot of work were done after that for this equation and other equations [12–33]. Following the works of Ablowitz [11, 13] and Fokas [12], we propose a two dimensional nonlocal nonlinear Schrödinger (NLS) equation

$$\begin{array}{c} \begin{array}{cc} & iu_{t}+u_{xx}+u_{yy}-2u_{xy}+2uV=0,V=u\left(x,y,t\right)u^{*}\left(-x,-y,t\right), \end{array} \end{array}$$ (2)

and a coupled nonlocal Klein-Gordon equation

$$\begin{array}{c} \begin{array}{cc} & u_{tt}\left(x,t\right)+u_{xx}\left(x,t\right)-\beta \;u\left(x,t\right)+\left[\epsilon u\left(x,t\right)u^{*}\left(-x,t\right)-2v\left(x,t\right)\right]u\left(x,t\right)=0,\\ & v_{tt}\left(x,t\right)-v_{xx}\left(x,t\right)+{\left[\epsilon u\left(x,t\right)u^{*}\left(-x,t\right)\right]}_{xx}=0, \end{array} \end{array}$$ (3)

here * means complex conjugation. For the two dimensional nonlocal NLS equation given by (2), this equation satisfies two dimensional fully *PT* symmetry condition *V*(*x*, *y*, *t*) = V*(−*x*, −*y*, *t*), where *q* is a complex function of *x*, *y*, *t*. Obviously, if select *V* = *u*(*x*, *y*, *t*)u*(*x*, *y*, *t*) in eq (2), the two dimensional nonlocal NLS equation reduces to a (2 + 1)-dimensional nonlinear Schrödinger equation in the Heisenberg ferromagnetic spin chain [34–40]. For the coupled nonlocal Klein-Gordon equation given by (3), although this equation is not invariant under *u*(*x*, *t*) → *u*(−*x*, −*t*), it has a conserved density *u*(*x*, *t*)u*(−*x*, *t*), which is invariant under spacial reversion together with complex conjugation as that for the nonlocal nonlinear Schrödinger equation (NLS), here *u*, *v* are functions of *x*, *t*. Corresponding to the nonlocal, the travelling wave transformation of the non-differentiable type of local equation is observed in [41–43].

The objective here is to demonstrate typical dynamics of breathers and rogue waves, intensively studied topics recently, which can be derived analytically for the two dimensional nonlocal NLS eq (2) and the coupled nonlocal Klein-Gordon eq (3) by employing the Hirota bilinear method [44]. Note that the Hirota bilinear method is an efficient and popular method to solve soliton equations [45–51]. In addition, the necessary conditions for the existence of solitary solutions of nonlinear partial differential equations are derived in [52, 53]. Breathers are pulsating modes and rogue waves are unexpectedly large amplitude displacements from a tranquil background [54]. Rogue waves were first observed in the oceans [54], but are now being pursued in optics and other fields as well [55–60]. In addition to the NLS equation, a variety of nonlinear soliton equations including nonlocal systems satisfied *PT* symmetry have been verified possessing rogue wave solutions [14, 15, 61–74]. Two recent articles [75] have provided a good review on the rogue waves from the physical view. Besides, as nonlinear wave interactions are important in the formation of different wave structures in physical systems, a good motivation of this paper is to derive different types of mixed solutions consisting of rogue waves, breathers and periodic line waves for the two dimensional nonlocal NLS eq (2) and the coupled nonlocal Klein-Gordon eq (3).

The outline of the paper is organized as follows. In Sect, three solutions of the two dimensional nonlocal NLS eq (2), namely, line breathers, rogue waves, semi-rational solutions consisting of line breather and rogue wave, are derived by employing the bilinear transformation method and taking a long wave limit, and their typical dynamics are analyzed and illustrated. In Sect, typical dynamics of several solutions for the coupled nonlocal Klein-Gordon eq (3), including rogue waves, breathers and mixed solution consisting of rogue waves, breathers, periodic line waves, are discussed. The Sect. contains a summary and discussion.

## Solutions of the two dimensional nonlocal NLS equation

The two dimensional nonlocal NLS equation is translated into the bilinear form

$$\begin{array}{c} \begin{array}{cc} & (iD_{t}+D_{x}^{2}+D_{y}^{2}-2D_{x}D_{y})g\cdot f=0,\\ & \left(D_{x}^{2}+D_{y}^{2}-2D_{x}D_{y}\right)f\cdot f=2\left[gg^{*}\left(-x,-y,t\right)-f^{2}\right], \end{array} \end{array}$$ (4)

through the variable transformation

$$\begin{array}{c} \begin{array}{c} u=e^{2it}\frac{g}{f}. \end{array} \end{array}$$ (5)

Here *f*, *g* are functions with respect to three variables *x*, *y* and *t*, and satisfy the condition

$$\begin{array}{c} \begin{array}{cc} & f^{*}\left(x,y,t\right)=f\left(-x,-y,t\right), \end{array} \end{array}$$ (6)

the asterisk denotes complex conjugation, and the operator D is the Hirota’s bilinear differential operator [44] defined by

$$\begin{array}{c} \begin{array}{cc} & P\left(D_{x},D_{y},D_{t},\right)F\left(x,y,t\cdots \right)\cdot G\left(x,y,t,\cdots \right)\\ = & P\left(\partial_{x}-\partial_{x^{{}^{\prime }}},\partial_{y}-\partial_{y^{{}^{\prime }}},\partial_{t}-\partial_{t^{{}^{\prime }}},\cdots \right)F\left(x,y,t,\cdots \right)G\left(x^{{}^{\prime }},y^{{}^{\prime }},t^{{}^{\prime }},\cdots \right){|}_{x^{{}^{\prime }}=x,y^{{}^{\prime }}=y,t^{{}^{\prime }}=t}, \end{array} \end{array}$$

where *P* is a polynomial of *D*_*x*_, *D*_*y*_, *D*_*t*_, ⋯.

By the Hirota’s bilinear method with the perturbation expansion [44], and take *f* and *g* be the forms of

$$\begin{array}{c} \begin{array}{cc} f= & \sum_{\mu =0,1}\exp\left(\sum_{k<j}^{\left(N\right)}\mu_{k}\mu_{j}A_{kj}+\sum_{k=1}^{N}\mu_{k}\eta_{k}\right),\\ g= & \sum_{\mu =0,1}\exp\left(\sum_{i<j}^{\left(N\right)}\mu_{k}\mu_{j}A_{kj}+\sum_{k=1}^{N}\mu_{k}\left(\eta_{k}+i\phi_{k}\right)\right), \end{array} \end{array}$$ (7)

then (5) produces the *N*-soliton solutions of the two dimensional nonlocal NLS equation. Here

$$\begin{array}{c} \begin{array}{cc} \eta_{j} & =iP_{j}\;x+iQ_{j}\;y+\Omega_{j}t+\eta_{j}^{0}\;,\\ \Omega_{j} & =\gamma_{j}\left(P_{j}-Q_{j}\right)\sqrt{4-{\left(P_{j}-Q_{j}\right)}^{2}}\;,\\ \exp(A_{jk}) & =\frac{{\left(P_{i}+P_{k}-Q_{i}-Q_{j}\right)}^{2}-2cos\left(\phi_{j}-\phi_{k}\right)-2}{-{\left(P_{i}+P_{k}-Q_{i}-Q_{j}\right)}^{2}-2cos\left(\phi_{j}+\phi_{k}\right)+2},\\ \cos(\phi_{j}) & =1-\frac{1}{2}{\left(P_{j}-Q_{j}\right)}^{2}\;,\sin\left(\phi_{j}\right)=-\frac{1}{2}\gamma_{j}\left(P_{j}-Q_{j}\right)\sqrt{4-{\left(P_{j}-Q_{j}\right)}^{2}}, \end{array} \end{array}$$ (8)

where *P*_*j*_, *Q*_*j*_ are freely real parameters, and *γ*_*j*_ = ±1. The natation ∑_*μ* = 0_ indicates summation over all possible combinations of *μ*_1_ = 0, 1, *μ*_2_ = 0, 1, …, *μ*_*N*_ = 0, 1; the $\sum_{j<k}^{N}$ summation is over all possible combinations of the *N* elements with the specific condition *j* < *k*.

**Remark 1.** The constraint (*P*_*j*_ − *Q*_*j*_)^2^ < 4 must hold for Ω_*j*_ to be real and |cos(*ϕ*_*j*_)|, |sin(*ϕ*_*j*_)| ≤ 1.

Following earlier works [14, 15, 76, 77] in the literature, a family of periodic solutions termed *n*th-order breathers can typically derived by taking parameters constraint

$$\begin{array}{c} \begin{array}{c} N=2n,P_{j+n}=-P_{j},Q_{j+n}=-Q_{j},\eta_{n+j}^{0}=\eta_{j}^{*0} \end{array} \end{array}$$ (9)

For example, taking parameters in (7)

$$\begin{array}{c} \begin{array}{c} N=2,P_{1}=-P_{2}=P,Q_{1}=-Q_{2}=Q,\eta_{2}^{0}=\eta_{1}^{*0}=\xi , \end{array} \end{array}$$ (10)

the first-order breather solution can also be expressed in terms of hyperbolic and trigonometric functions as

$$\begin{array}{c} \begin{array}{c} u=e^{2it}\frac{g_{2}}{f_{2}}, \end{array} \end{array}$$ (11)

where

$$\begin{array}{c} \begin{array}{cc} f_{2}= & \sqrt{M}\cosh\Theta +\cos\left(P\;x+Q\;y\right)\;,\\ g_{2}= & \sqrt{M}\left[\cos^{2}\phi \cosh\Theta +\sin^{2}\phi \sinh\Theta +i\cos\phi \sin\phi \left(\cosh\Theta -\sinh\Theta \right)\right]\\ & +\cos(P\;x+Q\;y)(\cos\phi +i\;\sin\phi )\;, \end{array} \end{array}$$ (12)

and

$$\begin{array}{c} \begin{array}{cc} & \exp\left(i\;\phi \right)=1-\frac{1}{2}{\left(P-Q\right)}^{2}-i\frac{1}{2}\left(P-Q\right)\sqrt{4-{\left(P-Q\right)}^{2}},\\ & M=\frac{4}{4-{\left(P-Q\right)}^{2}},\Omega =-\left(P-Q\right)\sqrt{4-{\left(P-Q\right)}^{2}},\\ & \Theta =\Omega \;\left(t-t_{0}\right),\exp\left(\Omega \;t_{0}\right)=\sqrt{M}\exp\left(\xi \right). \end{array} \end{array}$$

**Fig 1:**
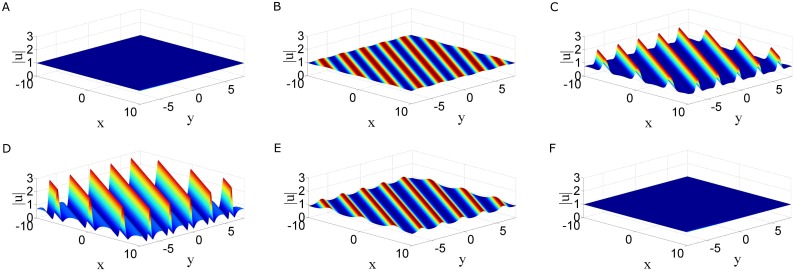
Time evolution of first-order breather solution. Time evolution of first-order breather solution |*u*| (11) of two dimensional nonlocal NLS eq (2) in (*x*, *y*) plane with parameters (13).

This solution for parameter choices

$$\begin{array}{c} \begin{array}{c} P=\frac{1}{2}\;,Q=\frac{1}{3}\;,\xi =0 \end{array} \end{array}$$ (13)

is shown in Fig 1. As can be seen, solution |*u*| given by (11) is the first-order line breather in the (*x*, *y*)-plane, which arises from the constant background possing profiles of parallel lines, and then decays back to the constant background again at larger time. The line breather is periodic in both *x* and *y* directions, and the period is $\frac{2\pi }{P}$ along *x* direction, while it is $\frac{2\pi }{Q}$ along *y* direction. The line breather has the characters: appearing from nowhere and disappear without a trace, which indicates that line rogue waves may exist in the two dimensional nonlocal NLS equation. Below, we consider rogue waves in two dimensional nonlocal NLS eq (2).

To generate rogue wave solutions of the two dimensional NLS equation, one can take a long wave limit of *f*_2_ and *g*_2_, i.e., take

$$\begin{array}{c} \begin{array}{c} \xi =i\pi \;,Q=\lambda \;P\;,P\to 0 \end{array} \end{array}$$ (14)

in eq (12), λ is an arbitrary real parameter, and λ ≠ 1. Then the first-order rogue wave solution of two dimensional nonlocal NLS eq (2) can be expressed in rational functions as

$$\begin{array}{c} \begin{array}{cc} u= & e^{2it}\left[1-\frac{\left(4it+1\right){\left(\lambda -1\right)}^{2}}{{\left(x+\lambda y\right)}^{2}+4{\left(\lambda -1\right)}^{2}t^{2}+\frac{1}{4}{\left(\lambda -1\right)}^{2}}\right]\;. \end{array} \end{array}$$ (15)

**Fig 2:**
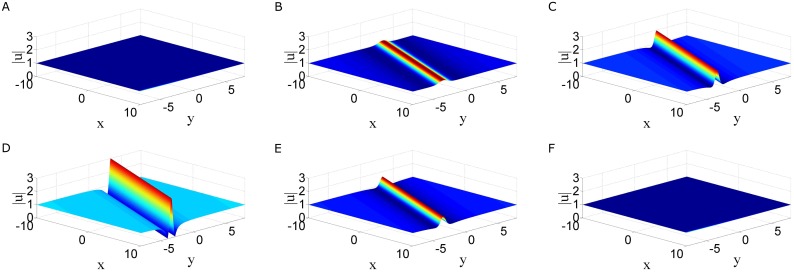
Time evolution of rogue waves in two dimensional nonlocal NLS equation. Time evolution of rogue waves (15) in two dimensional nonlocal NLS eq (2) in (*x*, *y*) plane with parameters λ = 3.

This rational solution has a line profile with a varying height (see Fig 2), and is different (2 + 1)-dimensional line solitons. Since the later maintains a perfect profile without any decay during their propagation in the (*x*, *y*)-plane. Besides, when *t* → ±∞, this solution |*u*| uniform approaches to the constant background 1; but in the intermediate time, |*u*| attains maximum amplitude 3 (i.e., three times the constant background amplitude) at the center of the line wave (*x* + λ*y* = 0) at *t* = 0. Hence this line wave describes the phenomenon: line waves appear from nowhere and disappear without a trace, and they are defined as line rogue waves [50, 51]. It is noted that the orientation of this line rogue wave is almost arbitrary as the parameter λ can be an arbitrary real parameters except 1. In particular, when one takes λ = 0 in the above line rogue wave, hence the solution *u* is independent of *y*. In this case, the two dimensional nonlocal NLS equation reduces to the one dimensional NLS equation, and this rogue wave of the two dimensional NLS equation reduces to the Peregrine rogue wave of the one dimensional NLS equation.

**Fig 3:**
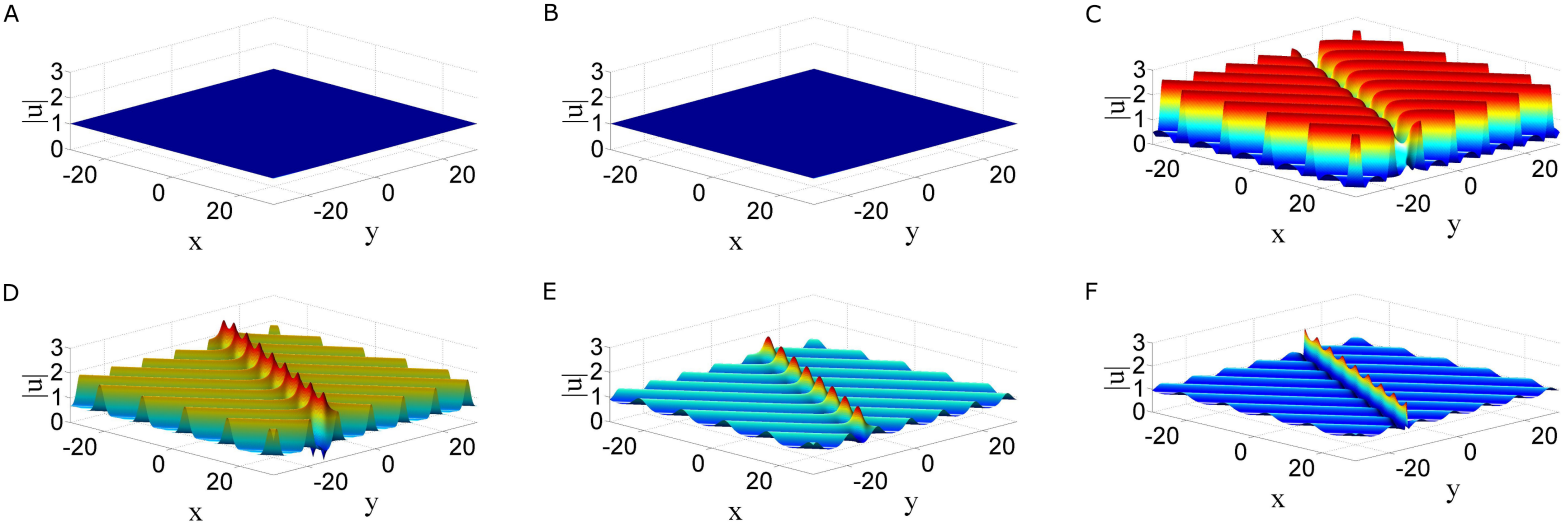
Time evolution of mixed solution |*u*| of the two dimensional nonlocal NLS equation. Time evolution of mixed solution |*u*| of the two dimensional nonlocal NLS eq (2) in (*x*, *y*) plane with parameters $\lambda_{1}=2,\lambda_{2}=2,P_{3}=\frac{1}{2},P_{4}=-\frac{1}{2},Q_{3}=-\frac{2}{3},Q_{4}=\frac{2}{3},\eta_{3}^{0}=0,\eta_{4}^{0}=0$.

We have discussed the breather solutions and rogue wave solutions respectively, below we derive a subclass of semi-rational solutions consisting of rogue waves and breathers. The simplest semi-rational solutions composed of one-breather and a fundamental line rogue wave can be generated from the fourth-order soliton. Indeed, taking parameters in (7)

$$\begin{array}{c} \begin{array}{c} N=4,Q_{1}=\lambda_{1}P_{1},Q_{2}=\lambda_{2}P_{2}=P,\eta_{1}^{0}=i\pi ,\eta_{2}^{0}=i\pi , \end{array} \end{array}$$ (16)

and then taking the limit as *P*_1_ → 0, *P*_2_ → 0, functions *f* and *g* of semi-rational solution *u* can be presented as

$$\begin{array}{c} \begin{array}{cc} f= & \theta_{1}\;\theta_{2}+a_{12}+\left(a_{14}\;a_{24}+a_{14}\;\theta_{2}+a_{24}\;\theta_{1}+\theta_{1}\;\theta_{2}+a_{12}\right)e^{\eta_{4}}+(a_{13}\;a_{23}+a_{13}\;\theta_{2}+a_{23}\;\theta_{1}\\ & +\theta_{1}\;\theta_{2}+a_{12})e^{\eta_{3}}+(a_{34}\;a_{13}\;a_{23}+a_{34}\;a_{13}\;a_{24}+a_{34}\;a_{13}\;\theta_{2}+a_{34}\;a_{14}\;a_{23}+a_{34}\;a_{14}\;a_{24}+\\ & a_{34}\;a_{14}\;\theta_{2}+a_{34}\;a_{23}\;\theta_{1}+a_{34}\;a_{24}\;\theta_{1}+a_{34}\;\theta_{1}\;\theta_{2}+a_{34}\;a_{12})e^{\eta_{3}+\eta_{4}}\\ g= & \left(\theta_{1}+b_{1}\right)\;\left(\theta_{2}+b_{2}\right)+a_{12}+(a_{14}\;a_{24}+a_{14}\;\left(\theta_{2}+b_{2}\right)+a_{24}\;\left(\theta_{1}+b_{1}\right)+\left(\theta_{1}+b_{1}\right)\;\left(\theta_{2}+b_{2}\right)\\ & +a_{12})e^{\eta_{4}+i\phi_{4}}+(a_{13}\;a_{23}+a_{13}\;\left(\theta_{2}+b_{2}\right)+a_{23}\;\left(\theta_{1}+b_{1}\right)+\left(\theta_{1}+b_{1}\right)\;\left(\theta_{2}+b_{2}\right)\\ & +a_{12})e^{\eta_{3}+i\phi_{3}}+(a_{34}\;a_{13}\;a_{23}+a_{34}\;a_{13}\;a_{24}+a_{34}\;a_{13}\;\left(\theta_{2}+b_{2}\right)+a_{34}\;a_{14}\;a_{23}+a_{34}\;a_{14}\;a_{24}\\ & +a_{34}\;a_{14}\;\left(\theta_{2}+b_{2}\right)+a_{34}\;a_{23}\;\left(\theta_{1}+b_{1}\right)+a_{34}\;a_{24}\;\left(\theta_{1}+b_{1}\right)+a_{34}\;\left(\theta_{1}+b_{1}\right)\;\left(\theta_{2}+b_{2}\right)\\ & +a_{34}\;a_{12})e^{\eta_{3}+\eta_{4}+i\phi_{3}+i\phi_{4}}, \end{array} \end{array}$$ (17)

where

$$\begin{array}{c} \begin{array}{cc} & \theta_{s}=ix+i\lambda_{s}y-2\left(\lambda_{s}-1\right)t,a_{12}=-\frac{1}{4}\left(\lambda_{1}-1\right)\left(\lambda_{2}-1\right),\\ & b_{1}=i\left(\lambda_{1}-1\right),b_{2}=i\left(\lambda_{2}-1\right),a_{34}=e^{A_{34}}\\ & a_{sl}=\frac{\left(P_{\ell }-Q_{\ell }\right)\left(\lambda_{s}-1\right)}{\sqrt{4-{\left(P_{\ell }-Q_{\ell }\right)}^{2}}-2}\;\left(s=1,2,\ell =3,4\right), \end{array} \end{array}$$ (18)

and *η*_*ℓ*_, *ϕ*_*ℓ*_ and $e^{A_{34}}$ are given by (8). Further, taking parameters constraints

$$\begin{array}{c} \begin{array}{c} \lambda_{2}=-\lambda_{1},P_{4}=-P_{3},Q_{4}=-Q_{3},\eta_{4}^{*0}=\eta_{3}^{*0}, \end{array} \end{array}$$ (19)

thus mixed solution composed of a fundamental line rogue wave and one line breather is generated. As can be seen in Fig 3, this solution approaches to the constant background as |*t*| >> 0. When a line rogue wave and one line breather arise arise from the constant background, the region of their intersection acquires higher amplitude first (see the panel at *t* = −2). Then the line breather rises to higher amplitudes in the intersection region, and the line rogue immerse into the line breather (see the panel at *t* = 0). At larger time, the breather decays back to the constant background with higher speed than the line rogue wave, and the line rogue wave surround by the breather appear on the constant background (see the panels at *t* = 1, 2). It is noticed that for all times, the maximum amplitudes of the line rogue wave do not exceed 3 (i.e., three times the constant background). As discussed that the maximum amplitude of the fundamental line rogue wave is three time the constant background amplitude, thus this interaction between the line rogue wave and the line breather does not generate very high peaks.

## Solutions of the coupled nonlocal Klein-Gordon equation

To using the Hirota bilinear method for constructing soliton solutions of the Eq (3), we consider a transformation different from that considered by Tajiri [78, 79]. Here we allow for nonzero asymptotic condition $\left(u,v)\to (\sqrt{2},\frac{\beta }{2}+\epsilon \right)$ as *x*, *t* → ∞, and look for solution in the form

$$\begin{array}{c} \begin{array}{c} u=\sqrt{2}\frac{\hat{g}}{\hat{f}},v=\frac{\beta }{2}+\epsilon -2{\left(\text{log}\hat{f}\right)}_{xx}, \end{array} \end{array}$$ (20)

where *f*, *g* are functions with respect to three variables *x*, *y* and *t*, and satisfy the condition

$$\begin{array}{c} \begin{array}{cc} & {\hat{f}}^{*}\left(-x,t\right)=\hat{f}\left(x,t\right). \end{array} \end{array}$$ (21)

Obviously, $u=\sqrt{2},v=\frac{\beta }{2}+\epsilon$ is a constant solution of the Eq (3), and under the transformation (20), the Eq (3) is cast into the following bilinear form

$$\begin{array}{c} \begin{array}{cc} & \left(D_{x}^{2}+D_{y}^{2}\right)\hat{g}\cdot \hat{f}=0,\\ & \left(D_{x}^{2}-D_{y}^{2}\right)\hat{f}\cdot \hat{f}=2\epsilon \left[\hat{g}{\hat{g}}^{*}\left(-x,t\right)-{\hat{f}}^{2}\right]. \end{array} \end{array}$$ (22)

We now solve the bilinear Eq (22) by taking $\hat{f}$ and $\hat{g}$ the forms of

$$\begin{array}{c} \begin{array}{cc} \hat{f}= & \sum_{\mu =0,1}\exp\left(\sum_{k<j}^{\left(N\right)}\mu_{k}\mu_{j}\hat{A_{kj}}+\sum_{k=1}^{N}\mu_{k}\hat{\eta_{k}}\right),\\ \hat{g}= & \sum_{\mu =0,1}\exp\left(\sum_{i<j}^{\left(N\right)}\mu_{k}\mu_{j}\hat{A_{kj}}+\sum_{k=1}^{N}\mu_{k}\left(\hat{\eta_{k}}+i\hat{\phi_{k}}\right)\right), \end{array} \end{array}$$ (23)

then (20) produces the *N*-soliton solutions of the coupled nonlocal Klein-Gordon equation. Here

$$\begin{array}{c} \begin{array}{cc} \hat{\eta_{j}} & =iP_{j}\;x+\Omega_{j}t+\eta_{j}^{0}\;,\\ \exp(\hat{A_{jk}}) & =-\frac{2\epsilon \cos\left({\hat{\phi }}_{j}-{\hat{\phi }}_{k}\right)+{\left(P_{j}-P_{k}\right)}^{2}+{\left(\Omega_{j}-\Omega_{k}\right)}^{2}-2\epsilon }{2\epsilon \cos\left({\hat{\phi }}_{j}+{\hat{\phi }}_{k}\right)+{\left(P_{j}+P_{k}\right)}^{2}+{\left(\Omega_{j}+\Omega_{k}\right)}^{2}+2\epsilon }, \end{array} \end{array}$$ (24)

with

$$\begin{array}{c} \begin{array}{cc} \Omega_{j} & =P_{j},\cos\left({\hat{\phi }}_{j}\right)=\frac{\epsilon -P_{j}^{2}}{\epsilon }\;,\sin\left({\hat{\phi }}_{j}\right)=\frac{\sqrt{2\epsilon -P_{j}^{2}}P_{j}}{\epsilon }, \end{array} \end{array}$$ (25)

or

$$\begin{array}{c} \begin{array}{cc} \Omega_{j} & =\sqrt{-P_{j}^{2}+4\epsilon },\;\cos\left({\hat{\phi }}_{j}\right)=-1,\sin\left({\hat{\phi }}_{j}\right)=0, \end{array} \end{array}$$ (26)

where *P*_*j*_ is an freely real parameters, and $\eta_{j}^{0}$ is an complex parameter.

**Remark 2.** The constraint $-P_{j}^{2}+4\epsilon \geq 0$ must hold for and $\hat{\Omega_{j}}$ to be real and $\left|\cos\left(\hat{\phi_{j}}\right)\right|,\left|\sin\left(\hat{\phi_{j}}\right)\right|\leq \;1$, thus hereafter we only discuss *ϵ* = 1.

**Fig 4:**
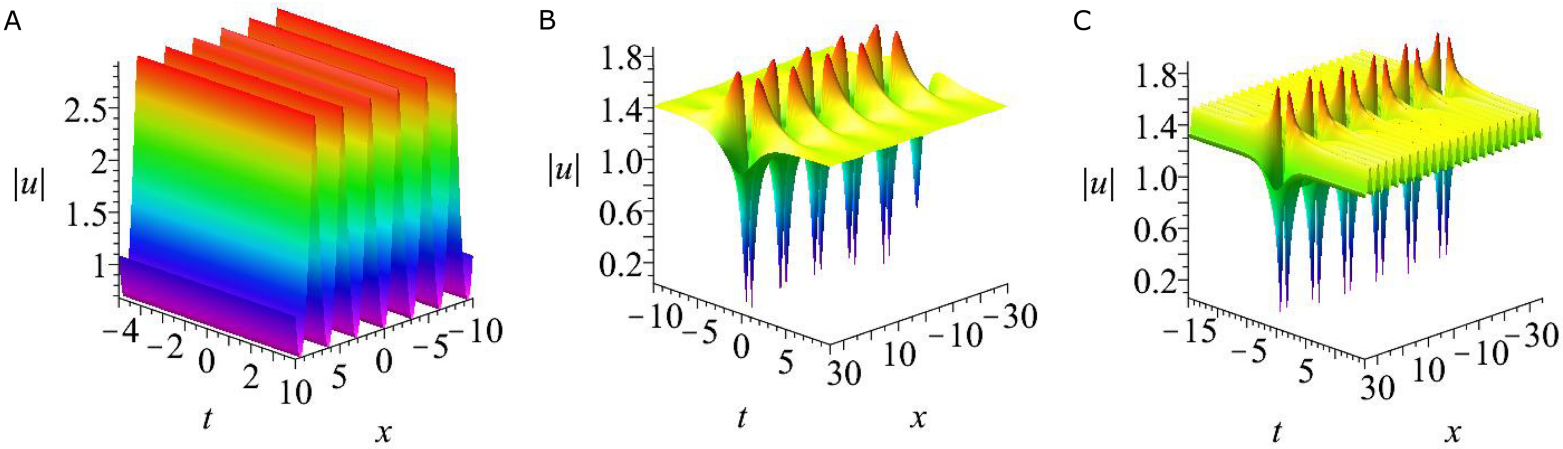
Three types of solutions for the coupled nonlocal Klein-Gordon equation. Three types of solutions for the coupled nonlocal Klein-Gordon eq (3). (a)Periodic line waves solution with parameters *N* = 1, *P*_1_ = 2, $\eta_{3}^{0}=0$, *ϵ* = 1. (b)Breather solution given by (29) with parameters (31). (c)A mixed solution consisting of a breather and periodic line waves with parameter (33) and $p_{1}=\frac{1}{2},p_{2}=-\frac{1}{2},\eta_{1}^{0}=0,\eta_{2}^{0}=0$.

In particular, when one takes *P*_*j*_ = ±2 in (26), the corresponding solutions are independent of *t*, thus they are periodic line waves which are localized in *t* direction, and the period is *π* along *x* direction, see Fig 4. A family of periodic solutions termed *n*th-order breathers can typically derived by taking parameters constraint in (23) and (25)

$$\begin{array}{c} \begin{array}{c} N=2n,P_{j+n}=-P_{j},\eta_{n+j}^{0}=\eta_{j}^{*0}. \end{array} \end{array}$$ (27)

For example, taking parameters in (23)

$$\begin{array}{c} \begin{array}{c} N=2,P_{1}=-P_{2}=P,\eta_{2}^{0}=\eta_{1}^{*0}=\xi , \end{array} \end{array}$$ (28)

the first-order breather solution can also be expressed in terms of hyperbolic and trigonometric functions as

$$\begin{array}{c} \begin{array}{c} u=\sqrt{2}\frac{{\hat{g}}_{2}}{{\hat{f}}_{2}},v=\frac{\beta }{2}+\epsilon -2{\left(\text{log}{\hat{f}}_{2}\right)}_{xx}, \end{array} \end{array}$$ (29)

where

$$\begin{array}{c} \begin{array}{cc} {\hat{f}}_{2}= & \sqrt{\hat{M}}\cosh\hat{\Theta }+\cos\left(P\;x\right)\;,\\ {\hat{g}}_{2}= & \sqrt{\hat{M}}\left[\cos^{2}\phi \cosh\hat{\Theta }+\sin^{2}\hat{\phi }\sinh\hat{\Theta }+i\cos\hat{\phi }\sin\hat{\phi }\left(\cosh\hat{\Theta }-\sinh\hat{\Theta }\right)\right]\\ & +\cos\left(P\;x\right)\left(\cos\hat{\phi }+i\;\sin\hat{\phi }\right)\;,\\ \hat{\Theta }= & \hat{\Omega }\;\left(t-t_{0}\right),\exp\left(i\;\hat{\phi }\right)=\left(1-\frac{P^{2}}{\epsilon }\right)+i\;\frac{P^{2}}{\epsilon }\sqrt{\frac{2\epsilon -P^{2}}{P^{2}}},\\ \hat{M}= & \frac{2\epsilon }{2\epsilon -P^{2}},\exp\left(\hat{\Omega }\;t_{0}\right)=\sqrt{\hat{M}}\exp\left(\xi \right). \end{array} \end{array}$$ (30)

This solution for parameter choices

$$\begin{array}{c} \begin{array}{c} P=\frac{1}{2}\;,\xi =0 \end{array} \end{array}$$ (31)

is shown in Fig 4(b). The corresponding solution is periodic in *x* direction and localized in *t* direction, the period is 4*π*.

Besides the breather solutions, a subclass of mixed solution consisting of periodic line waves and breather can also be generated from (23) by taking parameters

$$\begin{array}{c} \begin{array}{c} N=2n+1,P_{j+n}=-P_{j},\eta_{n+j}^{0}=\eta_{j}^{*0} \end{array} \end{array}$$ (32)

in (25) and *P*_2*n*+1_ = ±2, *η*_2*n*+1_ is defined in (26). For instance, taking parameters in (23)

$$\begin{array}{c} \begin{array}{c} N=3,P_{2}=-P_{1},\eta_{2}^{0}=\eta_{1}^{*0},P_{3}=2,\eta_{3}=2x+\eta_{3}^{0},e^{{\hat{\phi }}_{3}}=-1, \end{array} \end{array}$$ (33)

the corresponding solution is shown in Fig 4(c). It is seen that this solution is composed of a breather and periodic line waves. The period of the breather is $\frac{2\pi }{P}$ and the periodic line waves is 1.

To generate rogue wave solutions of the coupled nonlocal Klein-Gordon equation, we take a long wave limit of ${\hat{f}}_{2}$ and ${\hat{g}}_{2}$ in (30), i.e., take

$$\begin{array}{c} \begin{array}{c} \xi =i\pi \;,\epsilon =1,P\to 0 \end{array} \end{array}$$ (34)

in equation (30), then the first-order rogue wave solution can be expressed in rational functions as

$$\begin{array}{c} \begin{array}{cc} u= & \sqrt{2}\left[1-\frac{2\sqrt{2}it}{x^{2}+t^{2}+1}\right],\\ v= & \frac{\beta }{2}+1-\frac{4(x^{2}-t^{2}+1)}{{\left(x^{2}+t^{2}+1\right)}^{2}}. \end{array} \end{array}$$ (35)

The square of the short wave amplitude |*u*|^2^ has four critical points, namely,

$$A_{1}=\left(1,0\right),A_{2}=\left(-1,0\right),A_{3}=\left(0,1\right),A_{4}=\left(0,-1\right).$$

**Fig 5:**
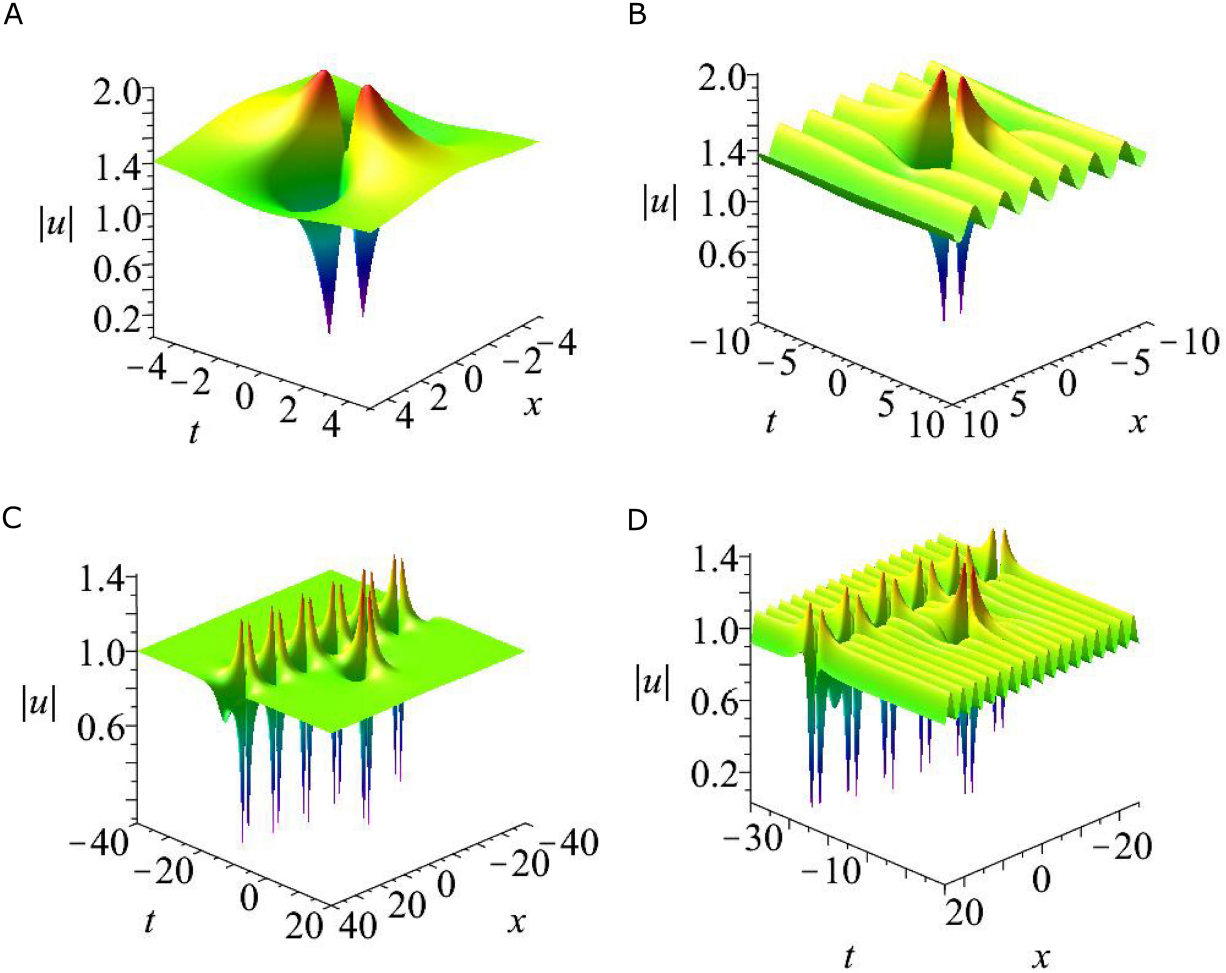
Four types of solutions for the coupled nonlocal Klein-Gordon eq (3). (a) Fundamental rogue wave solution |*u*| given by (35). (b) A mixed solution consisting of a rogue wave and periodic line waves with parameters (36). (c) A mixed solution consisting of a rogue wave and one breather with parameter $P_{3}=\frac{1}{2},P_{4}=-\frac{1}{2},\eta_{3}^{0}=2\pi ,\eta_{4}^{0}=2\pi$. (e) A mixed solution consisting of a rogue wave, one breather and periodic line waves with parameters $P_{3}=\frac{2}{3},P_{4}=-\frac{2}{3},p_{5}=2,\eta_{3}^{0}=\pi ,\eta_{4}^{0}=\pi ,\eta_{5}^{0}=0$.

Based on the analysis of critical points for rogue wave solutions (35), there are four-petaled rogue wave (i.e., two global maximum points *A*_1_, *A*_2_, and two global minimum points *A*_3_, *A*_4_) in the coupled nonlocal Klein-Gordon equation. The maximum value of |*A*| is 2 at points *A*_1_ and *A*_2_, while the the minimum value of |*A*| is 0 at the points *A*_3_ and *A*_4_. This fundamental rogue wave is illustrated in Fig 5(a).

Nonlinear wave interactions lead to several interesting dynamics in physical systems. Particularly, they are important in the formation of different wave structures. To show intriguing dynamical behaviour in the coupled nonlocal Klein-Gordon equation, we investigate three types of mixed solutions consisting of rogue waves, breather and periodic line waves.

**Type 1. A mixture of rogue wave and periodic line waves** We first consider the simplest semi-rational solutions, which are composed of a fundamental rogue wave and periodic line waves. Indeed, taking parameter choices in (23)

$$\begin{array}{c} \begin{array}{c} N=3\;,\epsilon =1,\eta_{1}^{0}=\eta_{2}^{0}=i\;\pi ,P_{3}=2,\eta_{3}^{0}=0,P_{1},P_{2}\to 0, \end{array} \end{array}$$ (36)

then functions $\hat{f}$ and $\hat{g}$ can be expressed as

$$\begin{array}{c} \begin{array}{cc} \hat{f}= & \left({\hat{\theta }}_{1}{\hat{\theta }}_{2}+{\hat{a}}_{12}\right)+\left({\hat{\theta }}_{1}{\hat{\theta }}_{2}+{\hat{a}}_{12}+{\hat{a}}_{13}{\hat{\theta }}_{2}+{\hat{a}}_{23}{\hat{\theta }}_{1}+{\hat{a}}_{12}{\hat{a}}_{23}\right)e^{{\hat{\eta }}_{3}}\;,\\ \hat{g}= & \left[\left({\hat{\theta }}_{1}+{\hat{b}}_{1}\right)\left({\hat{\theta }}_{2}+{\hat{b}}_{2}\right)+{\hat{a}}_{12}\right]+[\left({\hat{\theta }}_{1}+{\hat{b}}_{1}\right)\left({\hat{\theta }}_{2}+{\hat{b}}_{2}\right)+{\hat{a}}_{12}\\ & +{\hat{a}}_{13}\left({\hat{\theta }}_{2}+{\hat{b}}_{2}\right)+{\hat{a}}_{23}\left({\hat{\theta }}_{1}+{\hat{b}}_{1}\right)+{\hat{a}}_{12}{\hat{a}}_{23}]e^{{\hat{\eta }}_{3}+i{\hat{\phi }}_{3}}\;, \end{array} \end{array}$$ (37)

where

$$\begin{array}{c} \begin{array}{c} {\hat{\theta }}_{1}=ix+y,{\hat{\theta }}_{2}=ix-y,{\hat{a}}_{12}=-1,{\hat{a}}_{13}={\hat{a}}_{23}=-2,{\hat{b}}_{1}=-{\hat{b}}_{2}=i\sqrt{2}, \end{array} \end{array}$$ (38)

and ${\hat{\eta }}_{3},{\hat{\phi }}_{3}$ are given by (24) and (26). This solution describes an fundamental rogue wave on a background of periodic line waves, see Fig 5(b). Note that the maximum value of solution |*u*| is 2, which is the same with the maximum value of fundamental rogue wave solution |*u*| given by (35). Thus this interaction between the fundamental rogue wave and the periodic line waves does not generate higher peaks. That is different from the interaction between rogue waves and periodic line waves in the NLS equation, which can generate much higher peaks [12].

**Type 2. A mixture of rogue wave and breather** Another type of mixed solution is composed of a fundamental rogue wave and one breather, which can be generated from four-soliton solutions. Indeed, taking parameters in (23)

$$\begin{array}{c} \begin{array}{c} N=4,\eta_{1}^{0}=i\pi ,\eta_{2}^{0}=i\pi ,P_{4}=-P_{3},\eta_{4}^{*0}=\eta_{3}^{*0}, \end{array} \end{array}$$ (39)

and then taking the limit as *P*_1_ → 0, *P*_2_ → 0, functions $\hat{f}$ and $\hat{g}$ of semi-rational solutions *u* and *v* can be presented as

$$\begin{array}{c} \begin{array}{cc} \hat{f}= & {\hat{\theta }}_{1}\;{\hat{\theta }}_{2}+{\hat{a}}_{12}+\left({\hat{a}}_{14}\;{\hat{a}}_{24}+{\hat{a}}_{14}\;{\hat{\theta }}_{2}+{\hat{a}}_{24}\;{\hat{\theta }}_{1}+{\hat{\theta }}_{1}\;{\hat{\theta }}_{2}+{\hat{a}}_{12}\right)e^{{\hat{\eta }}_{4}}+({\hat{a}}_{13}\;{\hat{a}}_{23}+{\hat{a}}_{13}\;{\hat{\theta }}_{2}+{\hat{a}}_{23}\;{\hat{\theta }}_{1}\\ & +{\hat{\theta }}_{1}\;{\hat{\theta }}_{2}+a_{12})e^{{\hat{\eta }}_{3}}+({\hat{a}}_{34}\;{\hat{a}}_{13}\;{\hat{a}}_{23}+{\hat{a}}_{34}\;{\hat{a}}_{13}\;{\hat{a}}_{24}+{\hat{a}}_{34}\;{\hat{a}}_{13}\;{\hat{\theta }}_{2}+{\hat{a}}_{34}\;{\hat{a}}_{14}\;{\hat{a}}_{23}+{\hat{a}}_{34}\;{\hat{a}}_{14}\;{\hat{a}}_{24}+\\ & {\hat{a}}_{34}\;{\hat{a}}_{14}\;{\hat{\theta }}_{2}+{\hat{a}}_{34}\;{\hat{a}}_{23}\;{\hat{\theta }}_{1}+{\hat{a}}_{34}\;{\hat{a}}_{24}\;{\hat{\theta }}_{1}+{\hat{a}}_{34}\;{\hat{\theta }}_{1}\;{\hat{\theta }}_{2}+{\hat{a}}_{34}\;{\hat{a}}_{12})e^{\hat{\eta_{3}}+{\hat{\eta }}_{4}}\\ \hat{g}= & \left({\hat{\theta }}_{1}+{\hat{b}}_{1}\right)\;\left({\hat{\theta }}_{2}+{\hat{b}}_{2}\right)+{\hat{a}}_{12}+({\hat{a}}_{14}\;{\hat{a}}_{24}+{\hat{a}}_{14}\;\left({\hat{\theta }}_{2}+{\hat{b}}_{2}\right)+{\hat{a}}_{24}\;\left({\hat{\theta }}_{1}+{\hat{b}}_{1}\right)+\left({\hat{\theta }}_{1}+{\hat{b}}_{1}\right)\;\left({\hat{\theta }}_{2}+{\hat{b}}_{2}\right)\\ & +{\hat{a}}_{12})e^{{\hat{\eta }}_{4}+i{\hat{\phi }}_{4}}+({\hat{a}}_{13}\;{\hat{a}}_{23}+{\hat{a}}_{13}\;\left({\hat{\theta }}_{2}+{\hat{b}}_{2}\right)+{\hat{a}}_{23}\;\left({\hat{\theta }}_{1}+{\hat{b}}_{1}\right)+\left({\hat{\theta }}_{1}+{\hat{b}}_{1}\right)\;\left({\hat{\theta }}_{2}+{\hat{b}}_{2}\right)\\ & +{\hat{a}}_{12})e^{{\hat{\eta }}_{3}+i{\hat{\phi }}_{3}}+({\hat{a}}_{34}\;{\hat{a}}_{13}\;{\hat{a}}_{23}+{\hat{a}}_{34}\;{\hat{a}}_{13}\;{\hat{a}}_{24}+{\hat{a}}_{34}\;{\hat{a}}_{13}\;\left({\hat{\theta }}_{2}+{\hat{b}}_{2}\right)+{\hat{a}}_{34}\;{\hat{a}}_{14}\;{\hat{a}}_{23}+{\hat{a}}_{34}\;{\hat{a}}_{14}\;{\hat{a}}_{24}\\ & +{\hat{a}}_{34}\;{\hat{a}}_{14}\;\left({\hat{\theta }}_{2}+{\hat{b}}_{2}\right)+{\hat{a}}_{34}\;{\hat{a}}_{23}\;\left({\hat{\theta }}_{1}+{\hat{b}}_{1}\right)+{\hat{a}}_{34}\;{\hat{a}}_{24}\;\left({\hat{\theta }}_{1}+{\hat{b}}_{1}\right)+{\hat{a}}_{34}\;\left({\hat{\theta }}_{1}+{\hat{b}}_{1}\right)\;\left({\hat{\theta }}_{2}+{\hat{b}}_{2}\right)\\ & +{\hat{a}}_{34}\;{\hat{a}}_{12})e^{{\hat{\eta }}_{3}+{\hat{\eta }}_{4}+i{\hat{\phi }}_{3}+i{\hat{\phi }}_{4}}, \end{array} \end{array}$$ (40)

where ${\hat{\theta }}_{1},{\hat{\theta }}_{2},{\hat{b}}_{1},{\hat{b}}_{2}$ are given by (38), and

$$\begin{array}{c} \begin{array}{c} {\hat{a}}_{1j}=\sqrt{\frac{2P_{j}^{2}}{p_{j}^{2}-2}},{\hat{a}}_{2j}=-\sqrt{\frac{2P_{j}^{2}}{p_{j}^{2}-2}}\;\left(j=3,4\right),{\hat{a}}_{34}=e^{{\hat{A}}_{34}}, \end{array} \end{array}$$ (41)

and ${\hat{\eta }}_{s},{\hat{\phi }}_{s}\;(s=3,4,5),e^{{\hat{A}}_{34}}$ are given by (24) and (25). The corresponding solution is shown in Fig 5(c). It is seen that this solution consists of a rogue wave and a breather. This breather is still periodic in *x* direction and localized in *t* direction, the period is $\left|\frac{2\pi }{P_{3}}\right|$. It is noticed that altering the values of $\eta_{3}^{0}$, the location of the breather can be moved. For all the choices of $\eta_{3}^{0}$, the period of this breather does not have an visible change. That is different from this type of mixed solutions of the nonlocal NLS equation [13], as the latter has an unstable period.

**Type 3. A mixture of rogue wave, breather and periodic line waves** At the end of this section, we obtain a subclass of interesting mixed solutions consisting of a rogue wave, a breather and periodic line waves. This type of mixed solutions can be generated by taking a long wave limit of 5-soliton solutions. Taking parameters in (23)

$$\begin{array}{c} \begin{array}{c} N=5,\eta_{1}^{0}=i\pi ,\eta_{2}^{0}=i\pi ,P_{4}=-P_{3},P_{5}=2,\eta_{4}^{*0}=\eta_{3}^{*0}, \end{array} \end{array}$$ (42)

and then taking the limit as *P*_1_ → 0, *P*_2_ → 0, functions *f* and *g* of semi-rational solutions *u* and *v* are a combination of rational and exponential functions. Some interesting structures can be observed, see Fig 5(d). It is seen that both of the periodic line waves and the breather are periodic in *x* direction and localized in *t* direction. The period of the breather is $\left|\frac{2\pi }{P_{3}}\right|$, and the periodic line waves is 2. Although there are many researches about interactions between rogue waves and other types of nonlinear waves, but interactions between rogue waves, breathers and periodic line wave in 1 + 1 dimensions have not been reported before. Thus this type of semi-rational solution is a new solution.

## Summary and discussion

In this paper, we proposed two types of nonlocal soliton equations under *PT* symmetry conditions, namely, a two dimensional nonlocal NLS equation and a coupled nonlocal Klein-Gordon equation. By employing the Hiorta’s bilinear method, soliton and periodic line wave solutions were derived. Although these soliton solutions may have singularities, but smooth periodic line waves and breathers have been obtained by taking suitable choice of the parameters. For the two dimensional nonlocal NLS equation, line breathers are both periodic in *x* and *y* direction, see Fig 1. For the coupled nonlocal Klein-Gordon equation, breathers are localized in *t* direction and periodic in *x* direction, see Fig 4(b). In particular, a subclass of mixed solution consisting of breathers and periodic line waves is also generated see Fig 4(c). By taking a long wave limit of soliton solutions, the fundamental rogue wave solutions and semi-rational solutions have been generated. For the two dimensional nonlocal NLS equation, rogue wave solutions are line rogue waves, see Fig 2. The semi-rational solutions describe a line rogue wave and a line breather arising from the constant background together and then disappearing into the constant background again, see Fig 3. For the coupled nonlocal Klein-Gordon equation, except the rogue waves (see Fig 5(a)), semi-rational solutions describing the interactions between rogue waves, breathers and periodic line waves have also been generated. Three types of them are shown in Fig 5(b), 5(c) and 5(d). These nonlinear wave interactions lead to several interesting dynamics in physical systems, particularly, they are important in the formation of different wave structures. As there are few researches about the rogue waves of *PT*-symmetry systems, our research may help to promote the understanding of rogue wave phenomenon in *PT*-symmetry systems.
